# Resting Energy Expenditure and Metabolic Features in Children With Septo-Optic Dysplasia

**DOI:** 10.1210/jendso/bvaf031

**Published:** 2025-02-24

**Authors:** David J Cullingford, Jacqueline A Curran, Mary B Abraham, Aris Siafarikas, A Marie Blackmore, Jenny Downs, Catherine S Y Choong

**Affiliations:** Department of Endocrinology and Diabetes, Perth Children's Hospital, Nedlands, WA 6009, Australia; The Centre for Child Health Research, The Kids Research Institute Australia, University of Western Australia, Nedlands, WA 6009, Australia; Faculty of Health & Medical Sciences, University of Western Australia, Crawley, WA 6009, Australia; Department of Endocrinology and Diabetes, Perth Children's Hospital, Nedlands, WA 6009, Australia; Department of Endocrinology and Diabetes, Perth Children's Hospital, Nedlands, WA 6009, Australia; The Centre for Child Health Research, The Kids Research Institute Australia, University of Western Australia, Nedlands, WA 6009, Australia; The Centre for Child Health Research, University of Western Australia, Crawley, WA 6009, Australia; Department of Endocrinology and Diabetes, Perth Children's Hospital, Nedlands, WA 6009, Australia; The Centre for Child Health Research, The Kids Research Institute Australia, University of Western Australia, Nedlands, WA 6009, Australia; Faculty of Health & Medical Sciences, University of Western Australia, Crawley, WA 6009, Australia; Institute of Health Research, University of Notre Dame, Fremantle, WA 6160, Australia; School of Medicine & Health Sciences, Edith Cowan University, Mount Lawley, WA 6050, Australia; The Centre for Child Health Research, The Kids Research Institute Australia, University of Western Australia, Nedlands, WA 6009, Australia; The Centre for Child Health Research, The Kids Research Institute Australia, University of Western Australia, Nedlands, WA 6009, Australia; Curtin School of Allied Health, Curtin University, Bentley, WA 6845, Australia; Department of Endocrinology and Diabetes, Perth Children's Hospital, Nedlands, WA 6009, Australia; The Centre for Child Health Research, The Kids Research Institute Australia, University of Western Australia, Nedlands, WA 6009, Australia; Faculty of Health & Medical Sciences, University of Western Australia, Crawley, WA 6009, Australia

**Keywords:** septo-optic dysplasia, overweight and obesity, hypothalamic dysfunction, resting energy expenditure, hypopituitarism, hyperphagia

## Abstract

**Context:**

Septo-optic dysplasia (SOD) is a major cause of congenital hypopituitarism and is known to be associated with overweight and obesity in up to 44% of children. Given the role of the hypothalamus in hormonal regulation, we sought to assess the association of resting energy expenditure (REE), appetite and physical activity with SOD.

**Objective:**

To characterize REE and other metabolic features in patients with SOD and evaluate relationships with elevated body mass index (BMI).

**Methods:**

Children with SOD above 5 years of age attending Perth Children's Hospital participated. A CosMED Q-NRG indirect calorimeter was used to calculate mean measure REE (mREE). This was compared with predictive REE (pREE) based on the Schofield equation to determine mREE/pREE quotient. A BMI z-score >1 was considered elevated. Parents/carers completed a questionnaire about pituitary function, the Hyperphagia Questionnaire and the Sleep Disturbances Scale for Children (SDSC).

**Results:**

Twenty-six participants underwent testing (9 female, mean age 12.1 years) with 11 having elevated BMI and 15 with pituitary hormone deficiencies. Mean mREE was 1309 kcal/day (838-1732), mREE/pREE quotient was 88.8% ± 10.1. mREE/pREE quotient was similar in those with elevated BMI compared with normal BMI (83.3% ± 12.5 vs 92.1% ± 7.2, *P* = .068). Those with midline defects had a higher mREE/pREE quotient (91.8% ± 8.1 vs 80.4% ± 11.3, *P* = .026). Hyperphagia and SDSC scores were similar between BMI groups. Hyperphagia domain scores were higher in children with multiple hypopituitarism, pituitary structural defects, and normal septum pellucidum (*P* = .044, .042, and .033, respectively).

**Conclusion:**

Children with SOD had lower mREE than predicted and hyperphagia scores were higher in those with biochemical or structural pituitary changes, suggesting that hypothalamic dysfunction could drive BMI elevation in SOD. Indirect calorimetry may be used to guide the management of overweight and obesity in SOD.

Septo-optic dysplasia (SOD) is the most common congenital cause of multiple pituitary hormone deficiency (MPHD) [1], with international birth prevalence estimated to be approximately 1 in 10 000 and increasing [2-4]. SOD presents as a clinical triad, with a diagnosis based on the presence of 2 or more of the following: optic nerve hypoplasia (ONH), hypopituitarism, and midline defects (partial or complete absence/agenesis of the septum pellucidum or corpus callosum) [2]. The association relates to the embryological origin of the optic chiasm being immediately anterior to the developing pituitary, and the origin of the septum pellucidum and corpus callosum immediately posterior, in conjunction with the developing hypothalamus [5, 6]. An insult at this location can interrupt the development of these structures, resulting in SOD.

SOD has a highly variable phenotype with 55% to 80% of cases having pituitary hormone deficiency [7-10]. Additionally, there are high rates of overweight and obesity (up to 44%) [8, 9], with the etiology likely to be multifactorial. Due to its close anatomical relationship with other structures involved in SOD, and role in regulation of pituitary hormonogenesis, dysfunction of the hypothalamus is possible. Higher oral caloric intake is observed in mice with growth hormone (GH)–releasing hormone (GHRH) deficiency [11] and human subjects with GHRH receptor deficiency have greater daily intake [12] and higher ghrelin and hunger during and after a meal [13]. GHRH is produced by the hypothalamus to stimulate pituitary production of GH, and thus these hypothalamic models of GH deficiency support the interplay of hypothalamo-pituitary (HP) axis dysfunction and hyperphagia (excessive appetite). Children with intracranial tumors causing hypothalamic dysfunction frequently have hyperphagia and have been shown to have decreased resting energy expenditure (REE) [14, 15]. Children with SOD typically have some degree of visual impairment [16] and sleep disturbances may occur [17, 18], both of which may contribute to reduced physical activity, promoting weight gain [19, 20].

Measured REE in 67% of children at risk of hypothalamic dysfunction has been shown to be below the predicted range [14, 21]; however, to our knowledge this has not been tested in SOD. REE is the amount of kcal/day utilized by an individual at rest, a subset of total energy expenditure which includes additional expenditure due to diet-induced thermogenesis and physical activity. There are many formulae for predicting REE in adults and children, typically using height, weight, and gender, with specific age group cutoffs. The Schofield equation has been shown to perform most accurately in individuals above a healthy weight in the pediatric cohort [22]. Measured REE is performed through indirect calorimetry, where production of CO_2_ (VCO_2_) and oxygen consumption (VO_2_) are detected and utilized to determine the REE. Intraindividual variability in REE has been found to be minimal [23].

As the drivers of elevated body mass index (BMI) in SOD are not well-described, this study sought to delineate the REE and metabolic features. Specifically, we aimed to describe mean measure (m)REE in children and adolescents with SOD and compare with predictive (p)REE (Schofield equation), and compare clinical features, in particular participants with normal and elevated BMIs, hormonal deficiencies, and structural magnetic resonance imaging (MRI) changes, in terms of their REE, hyperphagia, sleep, and physical activity. Where data were available, we compared children and adolescents with SOD with other populations on these features.

## Materials and Methods

This was a pilot cross-sectional observational study conducted in children with SOD. The study was designed with input from a consumer reference group of parents/carers with an affected child.

Children and adolescents between 5 and 18 years of age, at the time of enrollment between April and August 2024 and currently or previously treated at the Child and Adolescent Health Service, Western Australia, were included in the study if they had (1) a diagnosis of SOD (as evidenced by 2 or more of the following: ONH or hypopituitarism or septum pellucidum/corpus callosum anomalies); (2) brain MRI data were available; (3) the diagnosis of SOD was made prior to December 31, 2023; and (4) parents/carers were willing for them to participate in REE measurements and/or complete a metabolic questionnaire. There were no exclusion criteria.

Patients were identified through endocrine outpatient clinics at the Perth Children's Hospital (PCH), the Western Australian Register of Developmental Anomalies (WARDA) database, and MRI records from the Department of Medical Imaging at PCH.

### Variables

Study visits were performed in the endocrinology outpatient clinic at PCH between 8:00 and 9:30 Am. During the study visit, height and weight were measured and used to calculate BMI and corresponding z-scores. Height, weight, sex, and age were recorded, and predicted (p)REE was derived using the Schofield equation. The COSMED Q-NRG Metabolic Monitor (COSMED, Rome, Italy) was used to perform indirect calorimetry measuring total kcal/day (mREE), VO_2_, VCO_2_, VO_2_/VCO_2_ variability. mREE was compared with pREE to determine percentage of pREE, described as the mREE/pREE quotient, with a low mREE/pREE quotient demonstrating overestimation of REE by the predictive equation. Testing was performed using the canopy hood system. Participants fasted for a minimum of 8 hours but allowed water and to take routine medication prior to testing. Tests were performed for 15 to 25 minutes (with the first 5 minutes excluded) and findings for the lowest variability 5-minute period were reported.

Fat mass and fat-free mass were calculated utilizing the Tanita Bc-420MA bioimpedance machine (Tanita Corp., Tokyo, Japan). Both the Tanita Bc 420MA and Cosmed Q-NRG devices are validated for children 5 years old and over. The Hyperphagia Questionnaire [24], Sleep Disturbances Scale for Children (SDSC) sleep initiation and maintenance and sleep disordered breathing domains [25], and the Children's or Youth Physical Activity Questionnaire (CPAQ or YPAQ) were completed during or close to the study visit. CPAQ was completed by parents/carers for children aged 5-11 years and the YPAQ by the adolescents for 12- to 17-year-old participants [26]. Parents/carers also reported their child's vision impairment and diagnosed hormonal deficiencies. All diagnoses of pituitary hormone deficiencies were made by a pediatric endocrinologist based on biochemical findings or provocative testing and treated with approved hormonal replacement. Adequacy of treatment with corresponding hormonal replacements was monitored by a pediatric endocrinologist with 3- to 6-monthly anthropometry, 3- to 6-monthly thyroxine levels, and 12-monthly measurement of insulin-like growth factor 1, and insulin-like growth factor binding protein 3, with dose adjustments accordingly.

Total hyperphagia score (11 questions, scored 1-5, total score 11-55, scores >19 consistent with hyperphagia [27]) and the Hyperphagia Questionnaire for Clinical Trials (HQCT) score, which is a subset of the hyperphagia questionnaire (9 questions, scored 0-4, total score 0-36), were calculated [28]. Metabolic equivalent of task (MET) scores corresponding to CPAQ and YPAQ findings were calculated to determine number of hours of moderate (MET scores 3-5.9) and vigorous (MET scores ≥6) physical activity. Based on these findings, attainment of “Australian 24-hour movement guidelines for children,” > 7 hours of moderate activity, and >3 hours of vigorous activity per week was recorded [29].

Ethics approval was obtained from the WA Department of Health, and Child and Adolescent Human Research Ethics Committee (RGS6516).

### Statistical Analysis

Descriptive statistics were used to characterize the study population. Elevated BMI was defined as ≥85th centile (BMI z-score ≥1), with obese being ≥95th centile (BMI z-score ≥1.65) and overweight being 85th to 95th centile (BMI z-score 1-1.64) [30]. Chi-square analysis was used for comparison of categorical variables and the Student t-test for continuous variables, using JASP version 0.19.1 (JASP, University of Amsterdam, Netherlands) data analysis software. Hyperphagia and sleep domain scores were compared with findings from other studies [25, 28, 31] using Stata version 18.0 (StatCorp, College Station, TX, USA) test of means function.

## Results

**Figure 1.**
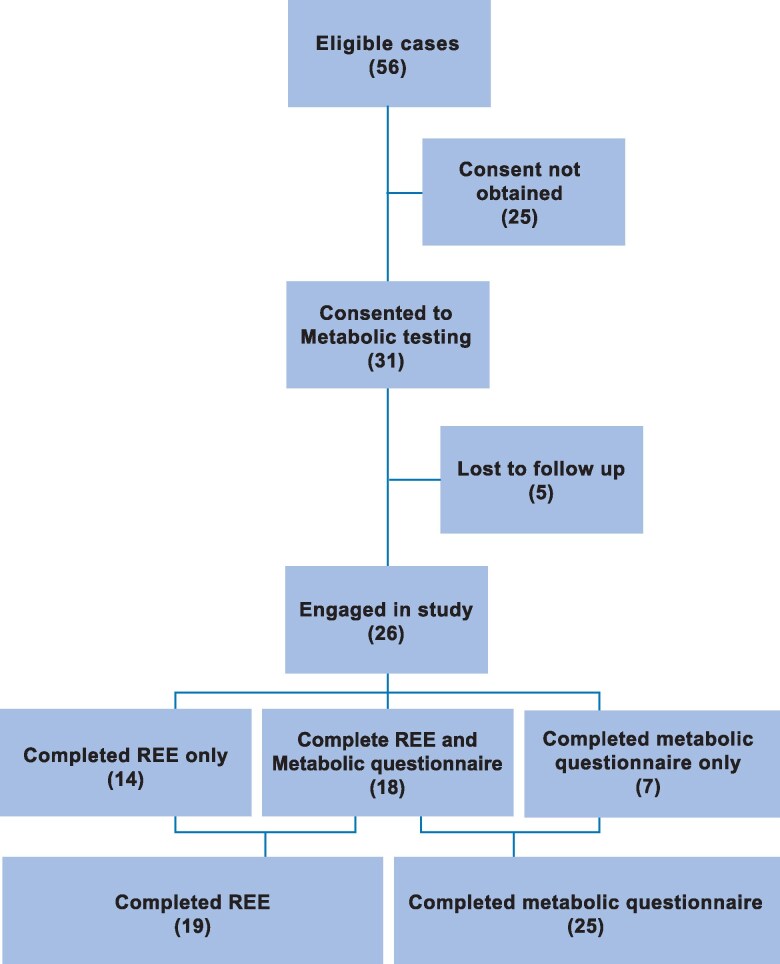
Enrollment of participants and description of groups used for analysis. Description of group with number of participants included in brackets.

Twenty-six children completed part or all of this study, with 19 successfully completing REE testing (1/20 was excluded after testing due to an insufficient fast) and 25 completing the questionnaire (Fig. 1). The mean age was 12.1 years (9 female) with mean BMI z-score of 0.39, and 11 having an elevated BMI (42.3%) with 7/26 having a BMI z-score 1-1.64 (overweight) and 4/26 having BMI z-score ≥1.65 (obese).

Vision impairment was reported in all but 1 child. Hypopituitarism was seen in 15/26 with 12 having MPHD. Each of these variables was similar for children whose BMI z-score ≥1 or <1. (Table 1).

**Table 1.** Cohort characteristics (n = 26)

	Total	BMI z-score ≥1	BMI z-score <1	*P*
Number	26	11	15	
Age (years)	12.1 ± 2.7	11 ± 2.5	12.9 ± 2.6	.08
Female	9/26	5/11	3/15	
BMI z-score	0.50 ± 1.27	1.71 ± 0.334	−0.38 ± 0.90	<.001
Height z-score	0.03 ± 1.16	0.25 ± 1.43	−0.131 ± 0.93	.412
Weight z-score	0.45 ± 1.21	1.51 ± 0.76	−0.33 ± 0.83	<.001
**Vision impairment**	25/26	10/11	15/15	.211
Severe	7/26	1/11	6/15	.132
**Pituitary deficiency**	15/26	7/11	8/15	.599
GH	11/26	6/11	5/15	.781
TSH	11/26	5/11	6/15	.279
ACTH	12/26	7/11	5/15	.126
FSH/LH	2/26	1/11	1/15	.819
ADH	7/26	4/11	3/15	.353
MPHD	12/26	7/11	5/15	.126
**Midline defects**	19/26	8/11	10/15	.973
Septum pellucidum	17/26	7/11	10/15	.873
Corpus callosum	6/26	3/11	3/15	.664
**Triad**	7/26	4/11	3/15	.353
**Pituitary defects**	16/26	7/11	9/15	.851
Anterior pituitary	8/26	2/11	6/15	.234
Posterior pituitary	15/26	7/11	8/15	.599
Pituitary stalk	13/26	5/11	8/15	.691
PSIS	8/26	2/11	6/15	.234
**Hyperphagia (n)**	25	10	15	
Hyperphagia score	17.5 ± 6.6	19.2 ± 8.0	16.3 ± 5.5	.296
Behavior (scores 5-25)	7.7 ± 3.5	9.0 ± 3.5	6.9 ± 3.4	.141
Drive (scores 4-20)	6.1 ± 2.6	6.9 ± 3.2	5.6 ± 2.0	.223
Severity (scores 2-10)	3.3 ± 1.5	3.3 ± 1.4	3.3 ± 1.6	.958
Hyperphagia (total >19)	6/25	3/10	3/15	.566
HQCT (0-36)	4.5 ± 5.5	6.0 ± 6.4	3.5 ± 4.7	.265
**Physical activity (n)**	22	9	13	
Moderate activity (hours/wk)	13.5 ± 13.7	**21.6 ± 17.1**	**7.9 ± 7.1**	.**017**
Vigorous activity (hours/wk)	5.7 ± 9.6	9.1 ± 14.0	3.3 ± 4.0	.173
MET vigorous activity goals	9/22	4/9	5/13	.779
MET moderate activity goals	17/22	6/9	11/13	.323
**Sleep (n)**	25	10	15	
Sleep DIMS (5-35)	15.2 ± 6.3	16.0 ± 6.1	14.6 ± 6.6	.597
Sleep SDB (3-15)	4.5 ± 1.7	4.4 ± 1.3	4.5 ± 2.0	.855

Bolding represents a difference (*P* < .05) between elevated BMI and BMI z-score <1 groups based on Student t-test.

Abbreviations: ACTH, adrenocorticotropin; ADH, antidiuretic hormone; BMI, body mass index; DIMS, disorders of initiating and maintaining sleep; FSH, follicle-stimulating hormone; GH, growth hormone; HQCT, Hyperphagia Questionnaire for Clinical Trials; LH, luteinizing hormone; MET, metabolic equivalent of task; MPHD, multiple pituitary hormone deficiency; PSIS, pituitary stalk interruption syndrome; SDB, sleep disordered breathing; TSH, thyroid stimulating hormone.

### Resting Energy Expenditure and Metabolic Assessment

Body composition was completed on 16 individuals, with fat % in the desired range for all cases with BMI z-score <1, and above the desired range in those with elevated BMI. The mean measured REE was 1316 kcal/day and the mean pREE was 1491 kcal/day with the measured REE 11.2% lower than pREE (*P* < .001). Table 2 presents mREE and the pREE values in both BMI subgroups. mREE was significantly lower than pREE based on the Schofield equation in both BMI subgroups. Although the mREE/pREE quotient was lower in the elevated BMI group, this did not reach statistical significance (*P* = .068).

**Table 2.** Resting energy expenditure findings (n = 19)

	Total	BMI z-score ≥1	BMI z-score <1	*P*
Number (gender)	19 (5 F/12 M)	7 (5 F/2 M)	12 (1 F/11 M)	
Age	12.6 ± 2.2	12.2 ± 1.7	12.8 ± 2.5	.564
**Anthropometry**				
BMI z-score	0.39 ± 1.22	1.66 ± 0.32	−0.35 ± 0.88	<.001
Height z-score	0.095 ± 1.17	0.71 ± 1.42	−0.26 ± 0.86	.079
Weight z-score	0.371 ± 1.29	1.64 ± 0.70	−0.37 ± 0.90	<.001
Weight (kg)	50.7 ± 14.5	62.4 ± 12.2	44.0 ± 11.2	.04
**Body composition (n)**	16	4	12	
Body fat %	19.0 ± 11.5	36.0 ± 4.4	13.3 ± 5.8	<.001
Muscle mass %	76.8 ± 10.8	60.7 ± 4.2	82.2 ± 5.5	<.001
**REE**				
mREE (kcal/day)	1316 ± 214	1311 ± 172	1320 ± 242	.94
Schofield pREE (kcal/day)	1491 ± 230	1590 ± 221	1433 ± 105	.068
mREE – pREE	−174 ± 166	−279 ± 207	−113 ± 105	.032
mREE/pREE quotient	**88.8% ± 10.1**	**83.3% ± 12.5**	**92.1% ± 7.2**	.068
mREE per kg (kcal/kg/day)	**27.5** ± 6.8	**21.4** ± 3.1	**31.1** ± 5.7	<.001
**Hormonal changes (mREE/pREE)**		**Deficient (treated)**	**Not deficient**	***P***
Any deficiency		**85.9% ± 10.9 (n = 10)**	**92.1% ± 8.6 (n = 9)**	.188
GH		**88.3% ± 10.4 (n = 6)**	**89.1% ± 10.4 (n = 13)**	.88
TSH		**85.3% ± 11.8 (n = 7)**	**90.9% ± 8.9 (n = 12)**	.26
ACTH		**83.2% ± 12.2 (n = 7)**	**92.1% ± 7.4 (n = 12)**	.062
ADH		**82.4% ± 13.3 (n = 6)**	**91.8% ± 7.1 (n = 13)**	.058
MPHD		**83.2% ± 12.2 (n = 7)**	**92.1% ± 7.4 (n = 12)**	.062
**Structural changes (mREE/pREE)**		**Defect**	**No defect**	***P***
**Midline defects**		**91.8% ± 8.1** *^a^* **(n = 14)**	**80.4% ± 11.3** *^a^* **(n = 5)**	.026*^a^*
Septum pellucidum		**91.3% ± 8.2 (n = 13)**	**83.4% ± 12.5 (n = 6)**	.115
**Pituitary defects (any)**		**86.8% ± 10.4 (n = 12)**	92.3% ± 9.3 (n = 7)	.27
Anterior pituitary		**86.7% ± 8.9 (n = 7)**	**90.1% ± 11.0 (n = 12)**	.49
Posterior pituitary		**86.9% ± 10.9 (n = 11)**	**91.4% ± 8.9 (n = 8)**	.35
Pituitary stalk		**84.8% ± 9.9 (n = 10)**	93.3% ± 8.9 (n = 9)	.068

Bolding represents a difference (*P* < .05) between mREE and pREE using the Student t-test.

Abbreviations: ACTH, adrenocorticotropin; ADH, antidiuretic hormone; BMI, body mass index; GH, growth hormone; MPHD, multiple pituitary hormone deficiency; REE, resting energy expenditure; TSH, thyroid stimulating hormone.

^*a*^Represents a difference in mREE/pREE quotient between cohorts (ie, elevated BMI and normal or affected and nonaffected groups) using the Student t-test, with *P* reported in the next column.

Participants with hypopituitarism on treatment had a mREE/pREE quotient (85.9% vs 92.1%, *P* = .188) similar to those without deficiency and no individual hormonal deficiency had a significant association with differences in mREE/pREE quotient. Those without a midline defect had a lower mREE/pREE quotient (80.6% ± 11.4 vs 91.9% ± 8.1, *P* = .026) than those with midline defects. No differences based on pituitary structural defects were observed.

### Hyperphagia

No differences in the total hyperphagia or HQCT scores between groups based on BMI were observed. Six of 25 had scores >19, which is consistent with hyperphagia, with 3 each from the normal and elevated BMI groups (Table 1). Table 3 demonstrates that MPHD and a normal septum pellucidum were associated with a higher hyperphagia drive score and presence of structural pituitary defects, particularly pituitary stalk defects, were associated with higher hyperphagia behavior scores.

**Table 3.** Metabolic features, pituitary function, and structural changes (n = 25)

	Hyperphagia behavior			Hyperphagia drive			Total hyperphagia score			Disordered sleep initiation		
	Unaffected	Affected	*P*	Unaffected	Affected	*P*	Unaffected	Affected	*P*	Unaffected	Affected	*P*
**Pituitary function**												
Hypopituitarism	6.9 ± 3.8 (n = 11)	8.4 ± 3.2 (n = 14)	.317	5.4 ± 2.0	6.6 ± 2.9	.26	16.3 ± 6.2	18.4 ± 6.9	.428	17.1 ± 5.6	13.6 ± 6.6	.18
MPHD	6.7 ± 3.4 (n = 14)	8.9 ± 3.4 (n = 11)	.137	**5.2** ± **1.8**	**7.3** ± **3.0**	.**044**	15.6 ± 5.7	19.9 ± 7.1	.103	15.5 ± 5.9	14.7 ± 7.0	.768
Diabetes insipidus	7.3 ± 3.7 (n = 19)	9.2 ± 2.6 (n = 6)	.256	5.8 ± 2.3	7.0 ± 3.3	.347	16.7 ± 6.6	20.0 ± 0.3	.292	**16.9** ± **6.2**	**9.7** ± **2.2**	.**011**
**Structural anomalies**												
Any pituitary anomaly	**6 ± 2.0** (n = 10)	**8.9 ± 3.9** (n = 15)	.**043**	5.3 ± 1.9	6.7 ± 2.8	.148	15.0 ± 3.7	19.0 ± 7.8	.162	17.6 ± 5.6	14.3 ± 6.4	.397
Anterior pituitary	7.8 ± 4.0 (n = 17)	7.6 ± 2.2 (n = 8)	.929	6.3 ± 2.6	6.0 ± 2.6	.877	18.0 ± 7.5	16.5 ± 5.0	.620	**17.6** ± **5.7**	**11.2** ± **5.0**	.**030**
Posterior pituitary	7.0 ± 3.8 (n = 11)	8.23 ± 3.3 (n = 14)	.376	5.6 ± 2.01	6.6 ± 2.9	.26	16.7 ± 6.4	18.1 ± 7.1	.623	17.8 ± 5.3	13.9 ± 6.4	.252
Pituitary stalk	**6.1 ± 1.9 (n = 12)**	**9.2 ± 4.0 (n = 13)**	.**022**	5.4 ± 1.7	6.8 ± 3.0	.145	15.1 ± 3.5	19.5 ± 8.2	.105	16.9 ± 5.2	14.3 ± 6.9	.493
Absent septum pellucidum	7.8 ± 3.6 (n = 9)	7.7 ± 3.6 (n = 16)	.952	**7.5** ± **2.4**	**5.4** ± **2.4**	.**033**	19.7 ± 5.4	16.2 ± 7.3	.221	14.7 ± 6.4	16.0 ± 6.3	.776

Bolding represents a difference (*P* < .05) between those with and without a change (affected and unaffected) using the Student t-test. Number affected noted by functional or structural change is noted in hyperphagia behavior column.

Abbreviation: MPHD, multiple pituitary hormone deficiency.

Subgroup analysis of HQCT scores for those aged 12-18 years were compared with findings from a cohort of 20 Australian adolescents with Prader–Willi syndrome (PWS) also aged 12-18 years from the Australasian Prader-Willi Syndrome Database [31]. HQCT scores in adolescents with SOD (n = 15) were significantly lower than children with PWS (5.2 **±** 5.5 vs 12.7 **±** 9.1; *P* = .004). HQCT scores were higher in adolescents with SOD than in typically developed adolescents (n = 112) from the United States (5.2 **±** 5.5 vs 2.3 **±** 3.3, *P* = .002) [28].

### Sleep

Sleep initiation and maintenance and sleep disordered breathing subscale scores were similar for each BMI group (Table 1). Higher sleep initiation and maintenance scores were observed with absence of anterior pituitary structural abnormalities or absence of diabetes insipidus, (*P* = .014 and .011 respectively) (Table 3).

When compared with the control sample (n = 1157) in the validation study for the SDSC questionnaire, both sleep initiation and maintenance, and sleep disordered breathing scores were higher than controls (15.1 ± 6.3 vs 9.9 ± 3.1 [*P* < .001] and 4.5 ± 1.9 vs 3.8 ± 1.4; *P* = .017), but similar to the clinical sample (n = 147) of those with a known sleep disorder (18 ± 6.9; *P* = .059 and 4.8 ± 2.6; *P* = .51) [25].

### Physical Activity

Attainment of physical activity goals was similar across BMI groups; however, those with an elevated BMI engaged in significantly more hours of moderate intensity activity than those with normal BMI (Table 1). No other parameters had significant differences in physical activity.

## Discussion

This cross-sectional study of children and adolescents with SOD demonstrated that REE scores are lower than predicted based on the Schofield equation, and that those without midline defects had a lower mREE/pREE quotient than those with midline defects. Overall, this supports that REE is reduced in SOD, and that this may be the result of underlying HP axis dysfunction. Hyperphagia drive scores were higher in subjects with MPHD or a normal septum pellucidum, and hyperphagia behavior scores were higher in those with pituitary structural defects, again suggesting an association between HP dysfunction and hyperphagia in SOD. Adolescents with SOD has a distinct hyperphagia phenotype with higher HQCT than developmentally typical adolescents but lower than PWS when compared with other studies. Sleep initiation and maintenance scores were lower in those with diabetes insipidus or differences of anterior pituitary structure. When compared with the validating study, scores were higher than in controls, but not different to those with a known sleep disorder, suggesting a predisposition to disordered sleep. Participants with an elevated BMI engaged in more moderate-intensity activity than those with normal BMI.

### REE and BMI Elevation

The low mREE/pREE quotient across the total cohort, including normal and elevated BMI groups, suggests that reduced REE is a potential driver for obesity in SOD. Overall, the findings support that REE is reduced in SOD, with a larger cohort required to determine if this is isolated to those with an elevated BMI, or across the population. Participants with elevated BMI were not demonstrated to have a lower mREE/pREE quotient than normal BMI counterparts based on the Schofield equation, although the trend of results suggested a potential difference. Alongside the small sample size limiting the capacity to distinguish between the 2 groups, other factors may also contribute. While up to 44% of pediatric cases of SOD may have an elevated BMI [8, 9], it is also common in the general population, as 26.2% of Western Australian children aged 5-17 have an elevated BMI [32]. Thus hypopituitarism and a low REE are not the only predisposing factors, with other biological, genetic, and social factors underlying the risk of increased BMI. Hyperphagia may also drive obesity in some cases, although this was not demonstrated in our cohort. Ultimately, lower REE may be driving the difference between normal and elevated BMI in some cases, but this remains unclear.

### Hypothalamic Dysfunction in SOD

SOD is a heterogenous condition brought together by its triad; however, it can lead to 2 distinct subgroups, those with ONH and hypopituitarism, or ONH and midline defects. Garcia-Filion et al found that absent septum pellucidum or corpus callosum did not confer an increased risk of hypopituitarism in children with ONH [33]. Lower mREE/pREE quotient seen in those with normal midline structures, who by diagnostic definition must have hypopituitarism, suggests that pituitary dysfunction, or its regulation by the hypothalamus, are likely drivers for lower REE scores across the cohort. Similar findings for individuals with MPHD or normal septum pellucidum having higher hyperphagic drive score, and higher hyperphagic behavior scores in those with pituitary structural defects further support involvement of the hypothalamus. This correlates with findings relating to interruption of GHRH function in murine and human studies resulting in increased intake and reduced satiety [11-13] and that dysfunction of the HP axis may contribute to hyperphagia. Demonstrating that hypothalamic dysfunction is a potential driver of the increased risk of elevated BMI in SOD is important because it suggests behavioral or pharmacological interventions may augment standard lifestyle interventions in its management.

### Treatment Opportunities

Measurement of REE within a multidisciplinary clinic, allows personalized management with targeted dietary and physical activity interventions. Muscle is more metabolically active than fat [34] and thus, alongside the role of physical activity in energy consumption, building of muscle mass may increase REE independently. Dietitian assessment and support can help target energy intake that is consistent with energy expenditure and is more effective at supporting BMI improvement than when based on pREE measurement [35]. In PWS, a condition with well-described hypothalamic dysfunction, behavioral interventions around clear expectations for the child about their menu, meal timing, and creating a consistent routine have been beneficial in preventing weight gain in those with hyperphagia [36, 37]. Regarding potential pharmacological interventions, glucagon-like peptide 1 agonists have shown promise in management of hypothalamic obesity [38, 39], as has setmelanotide (melanocortin-4 receptor agonist) [40], including in pediatric participants.

### Sleep Dysfunction

Sleep dysfunction is reported as a known feature in SOD but few studies have characterized this previously. Rivkees et al utilized actigraphy to demonstrate abnormal sleep rhythm in 6/19 ONH cases, with greater vision impairment, MPHD, and developmental delay more common in these cases [41]. While sleep problems are seen in 78% of cases of corpus callosum agenesis [42], these anomalies are seen in less than 30% of SOD cases. The findings that sleep initiation and maintenance and sleep-disordered breathing domain scores are higher than controls in other studies demonstrates that sleep dysfunction may affect many children with SOD and that further investigation in this area is needed.

### Limitations

The small sample size, reduced the availability of subgroup analysis, and absence of a local control group means that result comparisons have been drawn from other studies with participants from different backgrounds. The cohort had heterogenous hormonal deficiency profiles, and biochemical control of deficiency was not assessed, which may directly influence resting energy expenditure, although all participants requiring treatment were under the care of a pediatric endocrinologist with anthropometry and biochemical monitoring as described above. Physical activity findings were based on CPAQ and YPAQ questionnaires rather than by objective measurement, and no formal diet review to assess caloric intake was undertaken, limiting assessment of energy intake and expenditure between groups. Parental perception of vision was reported rather than objective visual acuity and thus correlation of vision impairment to other findings was limited.

### Future Directions

A multicenter study drawing a larger cohort is required to further delineate the REE characteristics of young people with SOD and its relation to BMI elevation, pituitary, and potentially hypothalamic dysfunction that have been suggested by our pilot study. Longitudinal studies assessing the effect of behavioral or pharmacological interventions would also assist ongoing management in the clinical space. Measurement of REE with indirect calorimetry for young people with SOD with elevated BMI as part of a multidisciplinary clinic, could support personalization of dietary and physical activity interventions, the efficacy of which could be monitored by repeat REE measurements.

## Conclusion

Lower REE scores than predicted across the cohort and higher hyperphagia domain scores in participants with hypopituitarism suggest that hypothalamic dysfunction is an important driver of increased rates of overweight and obesity in SOD. Differences in REE findings from predicted, particularly in those with elevated BMI, suggest that the Schofield equation is not sufficient to estimate REE in children with SOD and elevated BMI, and indirect calorimetry should be considered to support multidisciplinary management of the condition. The presence of hypothalamic dysfunction as a potential driver of elevated BMI in SOD suggests behavioral and pharmacological intervention may be efficacious, and further research is required to better delineate the drivers of obesity in SOD.

## Data Availability

The data that support the findings of this study are available on request from the corresponding author. The data are not publicly available due to privacy or ethical restrictions.

## Abbreviations

ACTH: adrenocorticotropin
ADH: antidiuretic hormone
BMI: body mass index
CPAQ: Children’s Physical Activity Questionnaire
DIMS: disorders of initiating and maintaining sleep
GH: growth hormone
GHRH: GH-releasing hormone
HP: hypothalamo-pituitary
HQCT: Hyperphagia Questionnaire for Clinical Trials
MET: metabolic equivalent of task
mREE: mean measure resting energy expenditure
MRI: magnetic resonance imaging
MPHD: multiple pituitary hormone deficiency
ONH: optic nerve hypoplasia
pREE: predictive REE
PSIS: pituitary stalk interruption syndrome
PWS: Prader–Willi syndrome
SDB: sleep disordered breathing
SDSC: sleep disturbances scale for children
SOD: septo-optic dysplasia
TSH: thyroid stimulating hormone
VCO_2_: production of CO2
VO_2_: oxygen consumption
YPAQ: Youth Physical Activity Questionnaire
